# Transcriptome profiling reveals expression signatures of cranial neural crest cells arising from different axial levels

**DOI:** 10.1186/s12861-017-0147-z

**Published:** 2017-04-13

**Authors:** Rachael Lumb, Sam Buckberry, Genevieve Secker, David Lawrence, Quenten Schwarz

**Affiliations:** 1 Centre for Cancer Biology, University of South Australia and SA Pathology, Frome Road, Adelaide, SA 5000 Australia; 2 University of Adelaide, Frome Road, Adelaide, SA 5000 Australia; 3 Harry Perkins Institute of Medical Research, Perth, WA 6008 Australia; 4 Australian Research Council Centre of Excellence in Plant Energy Biology, University of Western Australia, Perth, 6009 WA Australia; 5 ACRF Cancer Genomics Facility, Centre for Cancer Biology, SA Pathology, Adelaide, Australia; 6 School of Molecular and Biomedical Science, University of Adelaide, Adelaide, Australia

**Keywords:** Neural crest, Fate, RNA-seq, Neuropilin, Cranial neural crest

## Abstract

**Background:**

Cranial neural crest cells (NCCs) are a unique embryonic cell type which give rise to a diverse array of derivatives extending from neurons and glia through to bone and cartilage. Depending on their point of origin along the antero-posterior axis cranial NCCs are rapidly sorted into distinct migratory streams that give rise to axial specific structures. These migratory streams mirror the underlying segmentation of the brain with NCCs exiting the diencephalon and midbrain following distinct paths compared to those exiting the hindbrain rhombomeres (r). The genetic landscape of cranial NCCs arising at different axial levels remains unknown.

**Results:**

Here we have used RNA sequencing to uncover the transcriptional profiles of mouse cranial NCCs arising at different axial levels. Whole transcriptome analysis identified over 120 transcripts differentially expressed between NCCs arising anterior to r3 (referred to as r1-r2 migratory stream for simplicity) and the r4 migratory stream. Eight of the genes differentially expressed between these populations were validated by RT-PCR with 2 being further validated by *in situ* hybridisation. We also explored the expression of the Neuropilins (*Nrp1* and *Nrp2*) and their co-receptors and show that the *A-type Plexins* are differentially expressed in different cranial NCC streams.

**Conclusions:**

Our analyses identify a large number of genes differentially regulated between cranial NCCs arising at different axial levels. This data provides a comprehensive description of the genetic landscape driving diversity of distinct cranial NCC streams and provides novel insight into the regulatory networks controlling the formation of specific skeletal elements and the mechanisms promoting migration along different paths.

**Electronic supplementary material:**

The online version of this article (doi:10.1186/s12861-017-0147-z) contains supplementary material, which is available to authorized users.

## Background

Neural crest cells (NCCs) are a multipotent population of cells that arise from dorsal regions of the neural tube during early stages of embryonic development [1]. Due to their critical importance to a wide variety of tissues, deficiencies of NCCs underlie a highly prevalent group of congenital disorders termed neurocristopathies that include craniofacial anomalies and cardiac outflow tract defects [2]. Understanding the genetic programs controlling NCC development is therefore essential to provide insight to the origins and potential treatment of a large number of birth defects.

Different populations of NCCs are defined by the position at which they arise along the antero-posterior axis. Cranial NCCs arise anterior to somite 5 and give rise to bone, cartilage and tendons of the head, as well as sensory and sympathetic neurons of the peripheral nervous system. Vagal NCCs arise between somites 1–7 and give rise to the neurons and glia of the enteric nervous system and to cardiac NCCs which form vascular smooth muscle lining the pharyngeal arch arteries and also contribute to the aortic-pulmonary septum. Trunk NCCs arise posterior to the 4th somite and give rise to neurons and glia of the sensory and sympathetic nervous system, schwann cells and melanocytes [3].

Within these broad axially defined regions, NCCs can be further divided into sub-populations based on their migratory path and developmental fate. For example, cranial NCCs spanning the region between the mid-diencephalon and 5th somite are segregated into distinct migratory streams which mirror the transient segmentation of the neural tube into lineage-restricted units such as the diencephalon, midbrain and the hindbrain rhombomeres (r) [4, 5]. Cranial NCCs emigrating from each axial level follow distinct paths that drive segregation into distinct migratory streams that are maintained as these cells navigate the cranial mesenchyme. Thus, cranial NCCs arising anterior to r3, including those from the diencephaplon, midbrain and r1-r2, migrate into the frontonasal process, maxilla and first pharyngeal arch (PA1), whereas r4-derived NCCs migrate into PA2. NCCs populating these regions also give rise to specific structures such as Meckel’s cartilage, incus, malleus and trigeminal ganglia (Vth) in PA1, and middle ear ossicle and stapes, hyoid bone and facioacoustic ganglia (VIIth/VIIIth) in PA2 [6].

Migration of cranial NCCs within these distinct streams is under control of cell intrinsic and environmental cues that include several ligand-receptor pairs from the Eph/Ephrin [7–9], ERBB/Neuregulin [10], SDF/CXCR [11] and VEGFA/Semaphorin/Neuropilin signalling pathways [12–17]. Neuropilins (NRP1 and NRP2) are transmembrane co-receptors for guidance molecules of the class 3 semaphorin (SEMA3) family and for heparin binding isoforms of the vascular endothelial growth factor VEGFA [18]. During early stages of cranial NCC development NRP1 expression is restricted to NCCs within the r4 migratory stream while NRP2 is restricted to NCCs within the r1-r2 migratory stream [16]. Using an inducible Cre/LoxP lineage tracing system the *Nrp2* expressing cranial NCCs were further found to give rise to r1-r2 derived structures such as the trigeminal ganglia (Vth cranial ganglia) [19]. Mouse knockouts of *Nrp1* and *Nrp2* further demonstrate an essential requirement for these receptors in promoting migration of NCCs within different streams. Thus, r4-derived NCCs migrate aberrantly in *Nrp1* knockout mice, and r1-r2-derived NCCs migrate aberrantly in *Nrp2* knockout mice [12, 16]. In chick, *Nrp1* is also expressed by NCCs in the r4 migratory stream and controls migration toward VEGFA secreted by the surface ectoderm [20]. Although Neuropilins recruit signalling co-receptors such as the A-type plexins (PLXNA1-4) and VEGF receptors (VEGFR1-R2) to control axonal guidance [21], vascular growth [22] and motor neuron migration [23], the signalling co-receptors recruited in NCCs remain unknown.

Positional identity of NCCs along the antero-posterior axis is thought to be acquired prior to migration and to be under control of homeodomain transcription factors that promote segmentation and patterning of the rhombomeres from which the NCCs arise [4, 24, 25]. Thus, the unique combination of Homeobox (HOX) genes along the antero-posterior axis is likely to underlie the molecular differences of the distinct migratory streams. Indeed, distinct *Hox* expression profiles have also been identified in NCCs arising at different axial levels. However, as *Hox* expression in NCCs is under control of distinct enhancers, the *Hox* genetic code in NCCs differs from their original rhombomeric tissue [5, 24, 25]. While the distinct expression profiles of the *Hox* genes and Neuropilins demonstrate that NCCs of different migratory streams are molecularly distinct, the extent of these differences and the regulatory networks controlling their unique identity remain unknown.

Here we have uncovered the transcriptional profiles of cranial NCCs arising anterior to r3 (termed r1-r2 migratory stream) and r4 migratory streams by performing RNA sequencing (RNA-seq) on purified populations of cranial NCCs. Our RNA-seq, RT-PCR and *in situ* hybridisation analyses reveal many previously unappreciated transcripts showing differential expression between these distinct streams of cells. We also explored the expression of potential Neuropilin co-receptors and show that *A-type Plexins* are differentially expressed between these cranial NCC streams. Our analyses identify a large number of genes differentially expressed between cranial NCCs arising at different axial levels, providing a comprehensive resource for future analysis of these cellular populations.

## Results

### Isolation of cranial NCC streams

**Fig. 1:**
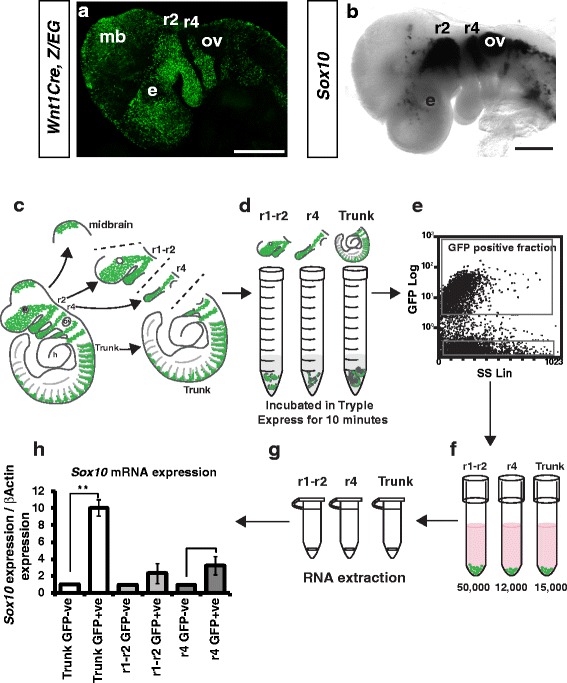
Isolation of GFP-positive NCCs. **a** Wholemount E9.5 *Wnt1Cre; Z/EG* embryos immunolabelled for GFP identifies NCCs in the r1-2 (r2) and r4 migratory streams, and additional *Wnt1* expression domains within the mid brain (mb). **b** *Sox10 in situ* hybridisation of wild type E9.5 embryos. **c**-**f** Schematic of work flow for isolation of GFP-positive NCCs from *Wnt1Cre; Z/EG* embryos. **c** *Wnt1Cre; Z/EG* embryos were dissected into r1-r2, r4 and trunk regions and dissociated in tryple express (**d**). **e** Trunk NCCs were used to set GFP gates for FACS. **f** GFP-positive NCCs were collected for each population and RNA extracted (**g**). **h** qRT-PCR was performed for *Sox10* to confirm NCC isolation. ov, otic vesicle; e, eye. Scale Bars = 500um

Previous studies have shown that NCCs arising anterior to r3 are molecularly distinct to NCCs within the r4 migratory stream [5, 19, 24, 25]. However, the extent of these differences has not previously been defined at the whole transcriptome level. To explore the transcriptional differences between these streams at embryonic day (E) 9.5 (i.e. embryos containing between 20 and 25 somites) when NCCs are actively migrating within the head and branchial arch tissue, we established a fluorescence activated cell sorting (FACS) technique to isolate NCCs from each stream. For this procedure we inter-crossed *Wnt1Cre* with *Z/EG* mice to permanently label all NCCs and their derivatives with green fluorescent protein (GFP) (Fig. 1a). As *Wnt1Cre* is also expressed in the midbrain (Fig. 1a) [26] we removed any GFP positive neural tissue prior to cell dissociation and FACS. Tissue anterior to r3 containing NCCs emigrating from the mid-diencephalon through to r1-r2, and tissue between r3 and r5 containing NCCs emigrating from r4 were dissected away from the trunk tissue under a dissecting fluorescent microscope (Fig. 1c-d). As these dissected regions contained only a small number of cells, tissue from GFP-negative littermates were added to boost cell numbers for FACS. GFP-positive NCCs were isolated from 4 individual *Wnt1Cre; Z/EG* litters (litter A, 7 GFP positive embryos; litter B, 4 positive embryos; litter C, 3 positive embryos; and litter D, 5 positive embryos). On average we isolated approximately 50,000, 12,000 and 15,000 GFP-positive cells from the r1-r2, r4 and trunk regions, respectively (Fig. 1e-f). Purification of GFP-positive NCCs was validated by qRT-PCR for *GFP* and the NCC marker *Sox10* (Fig. 1g-h). While this analysis showed enrichment of *Sox10* in the GFP-positive NCCs, expression was also observed in the GFP-negative cells as *Sox10* is expressed in NCCs from GFP-negative embryos used to boost cell numbers as well as non-NCC cell-types such as the otic vesicle (Fig. 1b).

### Dynamic expression of Neuropilins in cranial NCCs

**Fig. 2:**
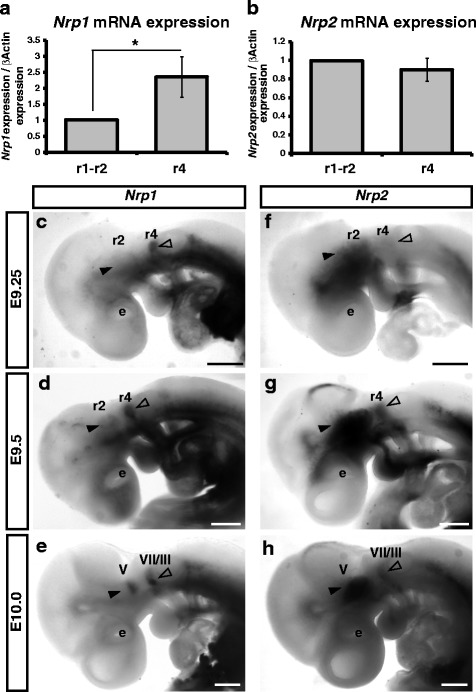
Expression of Nrp1 and Nrp2 in cranial neural crest cells. **a**-**b**) qRT-PCR for *Nrp1* (**a**) and *Nrp2* (**b**) in FACS isolated r1-r2 and r4 NCC streams. **a** *Nrp1* expression normalised to *β-Actin* in pooled data from four experiments is increased almost 2.5 fold (*N* = 4; *p* < 0.05). **b** *Nrp2* expression normalised to *β-Actin* in pooled data from four experiments is marginally increased in r1-r2-derived NCCs. **c**-**h** *In situ* hybridisation of *Nrp1* and *Nrp2* from E9.25-E10.0 demonstrates the complexity of Neuropilin expression. *Nrp1* is restricted to r4-derived NCCs at E9.25 (**c**, open arrow head). By E9.5 *Nrp1* remained highly expressed within r4-derived NCCs and at lower levels within r1-r2-derived NCCs (**d**, closed arrow head). **e** At E10.0 *Nrp1* is expressed in r4-derived facioacoustic ganglia (VII/VIII) and a subset of cells within the trigeminal ganglia (V). **f** *Nrp2* expression is restricted to r1-r2-derived NCCs at E9.25 (closed arrow head). **g** At E9.5 *Nrp2* expression is observed in both the r1-r2 and r4 migratory streams (open and closed arrow heads). **h** By E10.0 *Nrp2* is expressed in the developing trigeminal ganglia and within r4 NCC derivatives. e, eye. Scale Bars = 500um

Our previous expression analyses demonstrated that *Nrp1* and *Nrp2* are differentially expressed in r1-r2 and r4-derived cranial NCCs at E8.5-E9.5 [19]. Expression levels of *Nrp1* and *Nrp2* were therefore compared between NCCs from the r1-r2 and r4 migratory streams to validate that Neuropilins are indeed differentially regulated in these streams at E9.5. Consistent with our previous results [19] we found an increase of *Nrp1* expression in NCCs from the r4 migratory stream (Fig. 2a). However, there was no difference in the expression of *Nrp2* between r1-r2 and r4-derived NCCs (Fig. 2b). To clarify this unexpected result, we investigated the expression of *Nrp1* and *Nrp2* by *in situ* hybridisation from E9.25-E10.0. Consistent with our previous analysis we found that *Nrp1* expression was limited to r4-derived NCCs and that *Nrp2* expression was restricted to r1-r2-derived NCCs at E9.0-9.25 (Fig. 2c, f). At E9.5 when NCCs begin to differentiate and accumulate into the cranial ganglia, the expression of *Nrp1* remained strong within the r4 migratory stream and the anlagen of the facioacoustic cranial ganglia, and was also observed at lower levels within NCCs located in the r1-r2 migratory stream (Fig. 2d). However, while *Nrp2* expression remained high within the r1-r2 migratory stream and trigeminal ganglia at E9.5, we also observed expression within r4-derived NCCs (Fig. 2g). By E10.0 the expression of *Nrp1* and *Nrp2* was observed within NCC derivatives from both the r1-r2 and r4 migratory streams: *Nrp1* expression was expressed in a subset of cells within the trigeminal and facioacoustic ganglia, while *Nrp2* was observed within the r1-r2-derived trigeminal ganglia and within the facioacoustic ganglia (Fig. 2e-h). Taken together with our previous results, this analysis demonstrates that the expression of *Nrp1* and *Nrp2* are differentially expressed in the r1-r2 and r4 streams prior to E9.5, but soon thereafter become expressed in NCCs and NCC derivatives of both streams.

### RNA Sequencing reveals the expression profiles of r1-r2 and r4-derived NCCs

**Fig. 3:**
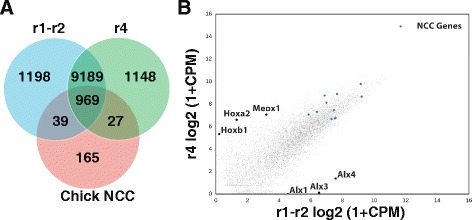
Transcriptome of r1-r2 and r4-derived NCCs. **a** Venn diagram illustrating the number of genes co-expressed in r1-r2 and r4-derived mouse NCCs compared to chick cranial NCCs. **b** Transcriptome analysis identified 34 genes enriched in r1-r2-derived NCCs (i.e. *Hoxa2*, *Hoxb1* and *Meox1*) and 87 genes enriched in r4-derived NCCs (i.e. *Alx1*, *Alx3* and *Alx4*). Several bona fide NCC genes are expressed abundantly in both populations of cells (*blue* dots represent *Crabp1*, *Snai1, Sox10*, *Foxd3, Sox9*, *Id1-3*, *Tfap2a, Ets1, Ngfr* and *Lin28*)

To determine the transcriptional profiles of NCCs in the r1-r2 and r4 migratory streams we performed RNA-seq on each purified population across 4 independent *Wnt1Cre; Z/EG* litters (litters A-D). Sequence generated from litters C and D had low mapping rates (litter A r1-r2 81%, r4 65%; litter B r1-r2 83%, r4 69%; litter C r1-r2 10%, r4 33%; litter D r1-r2 10%, r4 40%) and failed to cluster with other samples in principal component analysis (Additional file 1: Figure S1). While reduced mapping rates for the r4 stream indicate a small degree of contamination by non-murine cell types, expression profiles were normalised within and between samples by only using reads that map within mouse genes. By combining expression profiles of litter A and litter B we found that the r1-r2 and r4 streams had largely concordant expression profiles with 89% of genes showing similar expression in both samples (Fig. 3a and Additional file 2: Table S1) (criteria for expression taken as FPKM > 1). Within each sample we observed abundant expression of *bona fide* NCC markers, including *Crabp1*, *Snai1, Sox10*, *Foxd3, Sox9*, *Id1-3*, *Tfap2a, Ets1, Ngfr* and *Lin28* (Fig. 3b) [27, 28]. Indeed, we also found high concordance between our dataset and that of previous microarray analysis of mouse NCCs isolated from the first branchial arch and RNA-seq of chick cranial NCCs. Of 55 genes found to be expressed specifically in mouse NCCs of the first arch, we found 87% expressed within either the r1-r2 or r4 streams [29]. Moreover, 84% and 83% of genes up regulated in chick NCCs were also found in our datasets of r1-r2 and r4, respectively (Fig. 3a) [28].

Comparative expression analysis between r1-r2 and r4 identified a total of 121 genes meeting criteria for differential expression and included 34 genes up regulated in r1-r2 and 87 genes up regulated in r4 (see Tables 1 and 2 for the top 25 differentially expressed genes in r1-r2 and r4, and Additional file 2: Table S1 for a full list of genes). Gene ontology enrichment analysis of these results identified embryonic limb morphogenesis and skeletal development as the only over represented biological pathways for genes up-regulated in r1-r2 (*p* = 3.4E-04 and 9.84E-03, respectively), while response to retinoic acid was the only over represented biological pathway for genes up-regulated in r4 (*p* = 3.34E-03). Enrichment of genes within these biological processes fits well with the established roles of the retinoic acid pathway in NCC development [30] and the essential roles of NCCs in formation of the craniofacial skeleton [31]. Within the list of genes showing significant differences it is notable that *Alx1*, *Alx3* and *Alx4* are known to be up-regulated in NCC derivatives of the r1-r2 migratory stream, while *Hoxa2*, *Hoxb1* and *Ret* are also known to be expressed in NCCs of the r4 migratory stream [32–35]. Although not meeting criteria for significance, preferential expression of *Nrp1* and *Otx2* in either the r1-r2 or r4 migratory streams also fits well with previous findings [36, 37].

**Table 1:** Genes up-regulated in r1-r2-derived NCCs. Top 25 genes significantly up-regulated in the r1-r2 NCC migratory stream as defined via Cuffdiff and EdgeR. Inf refers to an infinite Log2 fold change due to a value of 0 in r4-derived NCCs

Gene id	r1-2	r4	log2	*p* value	q value	program
Nkx2-9	63.19	0.00	−12.55	7.77E-07	0.002	Both
Pax5	60.65	0.71	−7.83	4.83E-05	0.021	EdgeR
Sox21	55.40	3.19	−4.04	0.000115	0.034	EdgeR
Alx3	49.60	0.05	−9.01	2.38E-07	0.001	EdgeR
Shh	41.65	1.20	−5.67	9.06E-05	0.031	EdgeR
Alx4	34.33	0.30	−6.41	1.95E-07	0.001	EdgeR
Sp8	19.00	0.19	−7.52	6.77E-06	0.006	EdgeR
Trh	13.63	0.00	inf	0.00005	0.009	Cuffdiff
Ferd3l	13.06	0.00	inf	0.00005	0.009	Cuffdiff
Gm266	13.00	0.00	inf	0.00005	0.009	Cuffdiff
Icam4	11.47	0.00	inf	0.0003	0.042	Cuffdiff
Dleu7	10.64	0.00	inf	0.00005	0.009	Cuffdiff
Alx1	8.56	0.00	inf	0.00005	0.009	Cuffdiff
Polg2	8.32	0.00	inf	0.00005	0.009	Cuffdiff
Egr4	7.94	0.00	inf	0.00005	0.009	Cuffdiff
Sp9	7.70	0.00	inf	0.00005	0.009	Cuffdiff
Tmem28	5.78	0.00	inf	0.00005	0.009	Cuffdiff
Ncf1	5.32	0.00	inf	0.00005	0.009	Cuffdiff
Ccdc92	5.31	0.00	inf	0.00005	0.009	Cuffdiff
Hemk1	4.40	0.00	inf	0.00005	0.009	Cuffdiff
Hebp2	3.97	0.00	inf	0.00005	0.009	Cuffdiff
Gabre	3.26	0.00	inf	0.0001	0.017	Cuffdiff
Katnal2	3.11	0.00	inf	0.0002	0.031	Cuffdiff
Nr2e1	2.80	0.00	inf	0.00005	0.009	Cuffdiff
Ttll9	2.66	0.00	inf	0.0001	0.017	Cuffdiff
Sh2d5	2.54	0.00	inf	0.0003	0.042	Cuffdiff

**Table 2:** Genes up-regulated in r4-derived NCCs. Top 25 genes significantly up-regulated in the r4 NCC migratory stream as defined via Cuffdiff and EdgeR. Inf refers to an infinite Log2 fold change due to a value of 0 in r1-r2-derived NCCs

Gene id	r1-2	r4	log2	*p* value	q value	program
Hist1h2bm	38.20	28814.80	9.48	0.000112	0.034	EdgeR
Rprl2	25.60	22710.50	9.68	2.55E-06	0.004	EdgeR
Hist2h4	87.96	8685.48	6.63	0.00025	0.036	Cuffdiff
H2afj	17.84	6291.25	8.35	1.35E-05	0.010	EdgeR
Rmrp	21.77	5763.60	7.93	1.87E-05	0.012	EdgeR
Rpph1	5.23	2527.22	8.80	9.54E-06	0.008	EdgeR
Hist3h2a	6.03	2200.39	8.40	2.09E-05	0.012	EdgeR
Acta1	10.88	752.39	6.11	0.00015	0.024	Cuffdiff
Hist1h2bh	0.00	715.46	inf	0.00005	0.009	Cuffdiff
Rbp4	13.48	674.70	5.65	0.00025	0.036	Cuffdiff
Anxa2	1.52	543.21	8.36	2.85E-06	0.004	EdgeR
Afp	9.49	537.17	5.82	0.00015	0.024	Cuffdiff
Actc1	5.87	474.52	6.22	0.000129	0.034	EdgeR
Sparc	28.86	458.00	3.89	0.0002	0.049	EdgeR
Cav1	0.13	309.14	11.02	1.99E-06	0.004	EdgeR
S100a6	3.34	298.11	6.37	0.000124	0.034	EdgeR
Tgfbi	9.11	294.22	4.91	5.83E-05	0.023	Both
Krt14	0.29	286.73	9.84	1.02E-07	0.001	EdgeR
Hist1h3c	0.00	285.48	inf	0.00005	0.009	Cuffdiff
Efhd2	13.81	216.56	3.87	0.000128	0.034	Both
Ptrf	1.20	184.30	7.16	4.28E-06	0.004	EdgeR
Trnp1	1.21	174.43	7.06	8.76E-05	0.031	EdgeR
Cited4	0.22	155.37	9.28	6.87E-07	0.002	EdgeR
Des	3.64	142.70	5.18	1.50E-05	0.011	EdgeR
Serpine1	0.26	74.25	8.03	5.21E-07	0.002	EdgeR
Hoxa2	0.92	62.35	6.01	5.87E-06	0.006	EdgeR

### Validation of differentially expressed genes

**Fig. 4:**
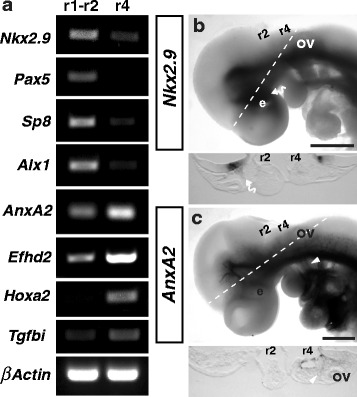
Validation of differentially expressed genes. **a** RT-PCR validation of genes significantly up regulated from r1-r2 and r4-derived NCCs. **b**-**c** Whole mount and sectioned *in situ* hybridisation of *Nkx2.9* (**b**) and *AnxA2* (**c**) validates RNAseq expression data. *Nkx2.9* and *is* observed within the periocular mesenchyme (curved arrows) populated by r1-r2-derived NCCs. *AnxA2* expression is observed within blood vessels and the second branchial arch which is populated by r4-derived NCCs (**c**, arrowhead). ov, otic vesicle; e, eye. Scale Bars = 500um

Transcriptomic analysis identified a number of genes differentially regulated between the r1-r2 and r4 migratory streams indicating that these populations are indeed different. To validate this data we performed semi-quantitative RT-PCR on cDNA from regions of r1-r2 and r4 dissected from wild type E9.5 embryos. Eight transcripts were selected from the differentially expressed genes identified from the analysis of litter A-B (Table 1, Table 2 and Additional file 2: Table S1). In comparison to *β-Actin* (*Actb*) all genes showed an expression pattern replicating the transcriptomic data, with *Alx1*, *Pax5*, *Nkx2.9* and *SP8* demonstrating higher expression in r1-r2, and *Hoxa2*, *AnxA2, Tgfb1* and *Efhd2* demonstrating higher expression in r4-derived NCCs (Fig. 4a). To further validate gene expression we selected two genes previously unknown to be expressed in NCCs and performed *in situ* hybridisation on E9.5 wild type embryos (Fig. 4b-c). *Nkx2.9* expression was observed in the floor plate of the neural tube where it has previously been implicated in the organisation and development of neural networks [38], and within periocular cranial mesenchyme (curved arrow, Fig. 4b). *AnxA2* expression was observed within blood vessels throughout the entire body and within the second branchial arch tissue, which is predominantly comprised of NCCs from r4 (arrowhead, Fig. 4c). RT-PCR and *in situ* hybridisation analysis therefore corroborates our transcriptomic analysis. Taken together, these studies identify a large number of genes differentially regulated in the r1-r2 and r4 populations of NCCs. To explore if our dataset could be used to identify regulatory networks promoting r1-r2 or r4 NCC identity we used oPOSSUM to search for transcription factor binding sites over-represented in the promoter regions of genes up-regulated in r1-r2 or r4 [39]. However, cross referencing of the top 20 over-represented transcription factors (Additional file 2: Table S2) against our list of significantly up-regulated data was unable to uncover any genes potentially acting as master regulators of these different streams.

### Differential expression of Neuropilin coreceptors in cranial NCCs

**Fig. 5:**
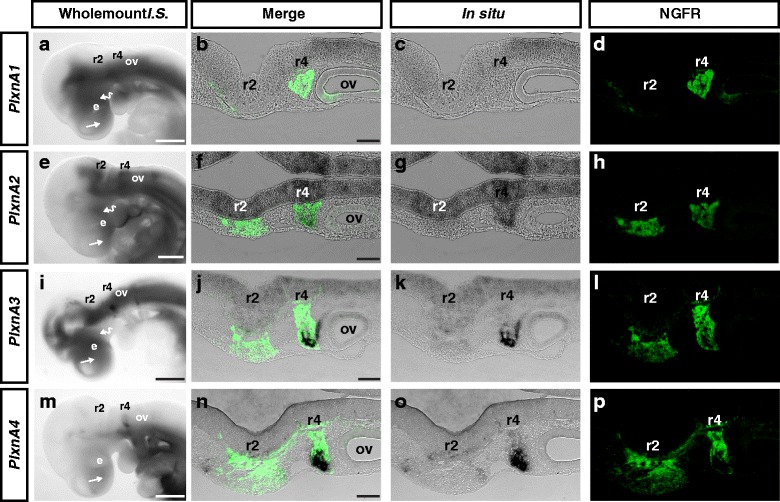
A-type Plexins are expressed within cranial NCCs. **a**-**p** Wholemount *in situ* hybridisation of wild type E9.5 embryos (**a**, **e**, **i**, **m**) were sectioned longitudinally (**b**-**c**, **g**-**f**, **j**-**k**, **n**-**o**) and immunolabelled for NGFR (**d**, **h**, **l**, **p**) to identify migrating NCCs. **a**-**e** *PlxnA1* is diffusely expressed throughout the mesenchyme of the frontonasal process, premaxilla and in the neural tube. **f**-**i** *PlxnA2* is expressed in the frontonasal process, premaxilla, diencephalon, rhombomeres and NCCs within the r1-r2 and r4 migratory streams. **j**-**m** *PlxnA3* is expressed in the frontonasal process, premaxilla and facioacoustic ganglia. **n**-**q** *PlxnA4* is expressed diffusely throughout the frontonasal process, premaxilla, PA1 and NCCs within the r4 migratory stream. ov, otic vesicle; e, eye. Scale Bars (**a**, **e**, **i**, **m**) = 500um and (**b**, **f**, **j**, **n**) = 100um

We next asked if the dataset could be used to identify putative NRP1 and NRP2 co-receptors controlling migration of the different migratory streams. Although PLXNA1-4 and VEGFR1-R2 are the archetypical Neuropilin co-receptors, several other receptors have been suggested to interact with Neuropilins or SEMA3, including PLXNB1-3, PLXNC1, PLXND1, L1CAM, NRCAM, Cd72, MET, ROBO1-4 and PTK7 [40–44]. Within our dataset we were unable to find expression of *VegfR1-R2*, *PlxnB3*, *Cd72* or *Robo1-3* which is consistent with previous reports and the known roles of these receptors in other cell types [22, 41–43]. Amongst the other putative Neuropilin co-receptors *PlxnA1*, *PlxnA3*, *PlxnA4*, *PlxnD1* and *Met* had differential expression in the r1-r2 and r4-derived NCCs. While a previous study has shown that *PlxnA3* and *PlxnA4* are not required for trunk NCC migration, their expression profiling raised the intriguing possibility that these receptors may be expressed in cranial NCCs [45]. We therefore investigated the expression of *PlxnA1-4* in the head by *in situ* hybridisation. At E9.5 *PlxnA1* was expressed diffusely throughout the entire embryo with highest expression in the frontonasal process, premaxilla and neural tube (Fig. 5a). Longitudinal sections through the head counterstained with antibodies specific for the p75 neurotrophin receptor (NGFR), a marker of NCCs, further demonstrated highest expression in the neural tube with lower expression in NCC and non-NCC mesenchyme (Fig. 5b-d). *PlxnA2* was expressed within the frontonasal process, premaxilla, diencephalon, hindbrain rhombomeres, r1-r2 and r4-derived NCCs (Fig. 5e-h). *PlxnA3* was expressed diffusely throughout the head mesenchyme and neural tube with heightened expression within the frontonasal process, premaxilla and facioacoustic cranial ganglia arising from NCCs in the r4 migratory stream (Fig. 5i-l). *PlxnA4* was expressed highest in NCCs within the r4 migratory stream and at lower levels in a subset of NCCs within the r1-r2 migratory stream (Fig. 5m-p). Taken together, our transcriptomic and *in situ* hybridisation analyses demonstrate that A-type Plexins are expressed in an overlapping pattern in cranial NCCs.

## Discussion

In this study we have used whole transcriptome profiling to reveal the genetic signatures of cranial NCCs arising from different axial levels. A large body of work in chick and zebrafish has previously defined gene regulatory networks that sequentially control: 1) specification of NCC precursors at the neural plate border, 2) specification of bona fide NCCs from the neuroepithelium, and 3) diversification of NCCs post delamination [46]. Under this model, a hierarchy of transcription factor combinations are proposed to drive different developmental stages, with neural crest specifier genes inducing formation of bona fide NCCs from their neuroepithelial precursors, and neural crest effector genes instructing NCC proliferation, migration and differentiation. Our current study shows that cranial NCCs from mice express a similar repertoire of specifier and effector genes to that seen in chick [27, 28, 46] and demonstrates that conserved genetic networks control NCC development across multiple species. In chick, SOX9 and ETS1 have also been shown to act as common enhancers of cranial NCC identity [28]. In further support of this notion, we identified SOX9 binding sites within the promoter regions of 77% and 82% of genes up-regulated in r4 and r1-r2, respectively.

Our findings also support the notion that different combinations of neural crest effector genes orchestrate the diverse developmental fates and migration paths of NCCs arising at different axial levels. Thus, our analysis identified distinct expression signatures for each NCC stream, including 10 transcription factors specific for r1-r2, and 14 transcription factors specific for r4-derived NCCs. Enrichment of the Aristaless-like homeobox gene transcription factors *Alx1*, *Alx3* and *Alx4* within r1-r2-derived NCCs is in strong agreement with previous findings linking these genes to early NCC development and craniofacial disorders [33, 47–50]. Moreover, identification of genes previously found enriched in NCCs arising anterior to r3 provides confidence that our dataset uncovers genetic networks underlying the diversity of NCCs arising at different axial levels. Amongst the other transcription factors specific to the r1-r2 stream *Ferd3l* has also been implicated in the craniofacial disorder Saethre-Chotzen syndrome [51], however, the remaining transcription factors have unknown roles in cranial NCCs. Sp8 and Sp9 are both members of the Sp/KLF transcription factor family that have essential roles in organising craniofacial, limb and interneuron development [52, 53]. Although Sp8 is primarily required by the anterior neural ridge and olfactory pit to regulate craniofacial development [54], it’s expression pattern is also consistent with a putative role in the frontonasal process at E9.5 in mice [52]. Expression of *Sp9,* on the other hand, is thought to be restricted to the ganglionic eminences and developing limb of mice post E10.5 [53]. While this is inconsistent with our data set, it is possible that prior analyses have not been sensitive enough to detect low levels of expression in NCCs at E9.5, as would be predicted by our data. The identification of *Sox21*, which is known to regulate neurogenesis, also suggests that our data set uncovers genetic networks at play in the differentiation phase of NCC development [55]. Indeed, this is further highlighted by the over-representation of genes regulating skeletal development in the r1-r2 data set, including *Alx1*, *Alx3*, *Alx4*, *Shh* and *Pax5*.

In contrast to NCCs that populate PA1, NCCs contributing to the second and more posterior pharyngeal arches are known to express various combinations of *Hox* genes. Accordingly, our analysis uncovered numerous *Hox* family members specific to r4-derived NCCs. *Hoxa2* was the most abundant member of this family and has well established roles in patterning second arch derivatives through regulating expression of effector genes such as Meox1 [56], which was also enriched in r4-derived NCCs. *Hoxb1* is exclusively expressed in r4 derived NCCs [32] and has essential roles in controlling formation of the facioacoustic ganglia [57]. *Hoxc11* and *Hoxc5* were enriched in r4-derived NCCs but have unknown functions in this stream. *Hoxc5* is predicted to have overlapping functions with its paralogue *Hoxb5* that has been shown to regulate expression of *Ret* in vagal NCCs [58]. *Ret* was also identified in our data set and previously shown to be enriched in r4-derived NCCs [34]. Another notable observation with the r4 data set is the over-representation of retinoic acid responsive genes including *Hsd17b2*, *Pparg*, *Abca1*, *Ret*, *Hoxa2* and *Rbp4*. Taken together with the finding that retinoic acid preferentially affects migration of r4-derived NCCs in chick [59], this suggests that this population of NCCs are more responsive to retinoic acid than NCCs arising anterior to r3.

Previous expression profiling and phenotypic analysis of *Nrp1* and *Nrp2* knockout mice supports the proposal that *Nrp1* guides r4-derived NCCs into PA2, and that *Nrp2* guides r1-r2-derived NCCs into PA1 [12, 16, 19]. Indeed, complete fusion of the trigeminal and facioacoustic ganglia in compound *Nrp1; Nrp2* knockout embryos highlights a critical role for these receptors in guiding neuroglial fated cranial NCCs [16]. While these receptors are expressed in exclusive domains at the earliest stages of NCC migration, our current analysis shows that they become co-expressed in the same populations of NCCs and NCC derivatives as they condense into the cranial ganglia. Taken in context, the knockout phenotypes suggest that the Neuropilins are required for promoting migration into distinct streams at the initial stages of NCC migration and in controlling axonal guidance after the cranial ganglia differentiate [16, 21].

Although none of the Neuropilin co-receptors were identified in our comparative transcriptome analysis, *in situ* hybridisation suggests that *PlxnA1-A4* are differentially expressed in NCCs arising at different axial levels. *PlxnA1-A4* are archetypical Neuropilin co-receptors that predominantly convey signals upon binding of SEMA3 during peripheral and central nervous system development [43]. Given their overlapping expression profiles it will now be of interest to address the roles of these receptors in controlling cranial NCC migration. In searching for additional mechanisms that may be involved in controlling cranial NCC migration it was notable that the receptor tyrosine kinase *Ret* and the GABA receptor *Gabre* were the only membrane receptors identified in our analysis. Ret is essential for migration of enteric NCCs within the gut but additional roles in controlling migration of r4-derived NCCs are currently unknown [60]. GABA receptors are also known to modulate the proliferation and differentiation of NCC derived boundary cap cells [61]. It will now be of interest to test if *Ret* or *Gabre* have specific roles in the r1-r2 or r4 population of NCCs.

## Conclusions

In conclusion, our studies uncover the transcriptional landscape that underpins diversity of cranial NCCs arising from different axial levels. This gene list provides novel insight to the regulatory networks controlling the formation of specific skeletal elements and to the mechanisms promoting migration along different paths.

## Methods

### Mice

All experimentation was approved by and conducted in accordance with the guidelines of the Animal Ethics Committee of SA Pathology/Central Adelaide Local Area Health Network and followed the Australian code of practice for the care and use of animals for scientific purposes. To obtain embryos of defined gestational ages, animals were mated in the evening, and the morning of vaginal plug formation was counted as embryonic day (E) 0.5. To label NCCs with GFP we crossed *Wnt1Cre* [62] mice to *Z/EG* mice [63]. All *Wnt1Cre* mice were maintained and used in the heterozygous state to minimise any off-target effects of aberrant expression of *Wnt1* that has been reported in this line [26].

### In situ hybridisation

Whole-mount *in situ* hybridisation was performed as described [23]. Riboprobes were transcribed from plasmids containing the cDNA sequence for *Nrp1* and *Nrp2* [64, 65]. Fragments of *Nkx2-9, Lmx1B* and *AnxA2* were amplified by PCR from whole embryo cDNA, cloned to pGEMT (Promega) and verified by sequencing. Primers used for PCR amplification are detailed in the RT-PCR methods section.

### Microdissection of NCC migratory streams

Somites were counted and GFP positive embryos with less than 25 somites were further dissected for fluorescent activated cells sorting (FACS). To isolate pure populations of r1-r2 and r4-derived cranial NCCs these regions were carefully dissected by first removing the GFP expressing forebrain which was discarded. The vagal and trunk NCCs were then removed by slicing the embryos through the otic vesicle. Finally, the r1-r2 and r4 streams were sliced apart using 26 gauge needles. GFP negative littermates were sliced into fragments and added evenly across the dissected r1-r2, r4 and trunk regions to boost cell numbers for further analyses.

### FACS sorting primary NCCs

Primary NCCs were isolated from E9.5 *Wnt1Cre; Z/EG* embryos as previously described [66]. To dissociate the dissected tissue Tryple Express (Life Technologies) was warmed to 37°C and 2mls added per tube. Tissue was incubated at room temperature for 10mins. Cells were gently triturated with a fire blown glass pipette until no clumps of cells could be seen, washed twice with Dulbecco’s modified Eagle’s media (DMEM) containing 1% Fetal Calf Serum (FCS) and strained through a 40μm filter. Cell sorting was performed on Beckman Coulter Epics Altra HyperSort using Expo MultiComp Software version 1.2B (Beckman Coulter) equipped with Innova 300C water-cooled 488nm argon laser at 100mW. Sorting was conducted at room temperature, with the instrument pressurised to 12psi and a 100um nozzle. Linear forward scatter (FSC) height (pk), width (TOF) and area (INT) signals were collected to allow for standard scatter and doublet discrimination. Linear side scatter (SSC) area (INT) signal was collected with a 488/10 band pass filter in PMT1. Log GFP signal was collected in PMT2 with a 525/25 band pass filter behind a 488nm long pass dichroic mirror. A gate was drawn on a FSC (INT) vs SSC (INT) plot to exclude debris and dead cells as discriminated by scatter properties alone. Following this a FSC pk vs FSC INT plot was examined to allow distinction of single cells. Linearly related cells were gated for further analysis on a GFP vs SSC plot. Cells were collected into DMEM with 1% FCS.

### RNA extraction

FACS isolated cells were sorted into DMEM containing 1% FCS, pelleted and then resuspended in 500ul of trizol before being frozen at −80°C. Upon thawing 100ul of chloroform was added and the samples mixed. Samples were centrifuged at 14,000rpm. The top aqueous phase was transferred into a new tube and an equivalent volume of ethanol was added and gently mixed. RNA was extracted from the ethanol mixed samples using a RNeasy Micro Kit (Qiagen). Half of the extracted RNA (6ul) was used for qRT-PCR and half used for RNAseq.

### Quantitative RT-PCR

Single stranded cDNA was made using a Quantitect Reverse Transcription kit (Qiagen). qRT-PCR was performed with SYBR Green reagent (Qiagen) using Rotor-Gene-6000 real-time PCR system (Corbett Life Science). Semi-quantitative PCR was also performed for a number of genes to compare the relative expression levels in the different NCC sub-populations. For this, go taq green (Promega) was used with a low number of cycles ranging from 20 to 25 and annealing temperature of 55°C. Primers used were as follows: *Sox10* fwd: GGAGGCAGAATGCCCAGGCG, rev: TGGCTCTGGCCTGAGGGGTG; *Nrp1* fwd: AAAGGTTCCTCCAATTGCTG, rev: TGGCTTCCTGGAGATGTTCT*; Nrp2* fwd: TGCATGGAGTTCCAGTACCA, rev: CCCTATCACTCCCTCGAACA*; β-Actin* fwd: GATCATTGCTCCTCCTGAGC, rev: GTCATAGTCCGCCTAGAAGCAT*; Sp8* fwd: GCGCACACTTGCACCATATC, rev: GTTCTTCTCGCGTTCCCCTT; *Hoxa2* fwd: CCTTTTGAGCAGACCATTCC, rev: AAAGCTGAGTGTTGGTGTACG; *AnxA2* fwd: CTTCAAGGGAGGCTCTCAGC, rev: GTAGAATGATCACCCTCCAGGC, *Nkx2-9 Fwd:* GCGCAGCCTCCTGAATTTAC, rev: TCTCGTCCGAGGACAGGTAG; *Alx1* fwd: CAAGTGGAGAAAAAGAGGAACG, rev: ATTCTGGTGGTTCGAAAACC; *Pax5* fwd: CTGTGACAATGACACTGTGC, rev: ACTGATGGAGTATGAGGAGC; *Tgfbi* fwd: ACAAACTGGAAGTCAAGCTCG rev: CTAATGCTTCATCCTCTCCAG; *Efhd2 fwd:* GATTTCGACAGCAAACTCAGC, rev: GAAAGTAGCTGGTACCAAAGG; *GFP* fwd: GCACGACTTCTTCAAGTCCGCCATGCC rev: GCGGATCTTGAAGTTCACCTTGATGCC. Relative mRNA levels were quantified using the comparative quantitation method in the Rotor-Gene −6000 software. Relative mRNA levels were then normalised to *β-Actin.* Each PCR was performed in triplicate across 4 biological replicates. Error bars represent standard error of the mean (SEM) between biological replicates.

### Immunostaining

Embryos were fixed in 4% paraformaldehyde in PBS. Sections were cut to a thickness of 18μm on a CM1850 cryostat (Leica, North Ryde, NSW, Australia) and air dried for a minimum of 60min prior to staining. For immunolabelling, whole-mount or sections were blocked in PBS containing 0.2% BSA and 0.5% Triton X-100 and stained with indicated primary antibodies: chicken anti-GFP 1:1000 (Sigma Aldrich, Sydney, NSW, Australia), rabbit anti-P75-NTR/NGRF 1:200 (Epitomics, Burlingame, CA, USA).

### RNA sequencing

RNA was enriched for polyadenylated transcripts before library construction with NEBNext RNA library preparation kit and sequenced on an Illumina HiSeq 2500 at the ACRF Cancer Genomics Facility (SA Pathology, Adelaide, Australia) to obtain 51 base single-end reads. Reads were trimmed for the NEB single end adapter “AGATCGGAAGAGCACACGTCTGAACTCCAGTCAC” with Cutadapt v1.3 [67], requiring a minimum overlap of 5, allowing a 20% error rate and discarding trimmed sequences shorter than 18 bases. The trimmed reads were then mapped to the UCSC mm10 mouse genome with Tophat 2.0.9 [68] using default parameters. Differential expression between r1-r2 and r4 was performed using Cuffdiff v2.1.1 or edgeR. For edgeR analysis, gene counts were obtained with HTSeq-count v0.6.1p1, and differential expression performed according to the protocol described in Anders 2013. Genes with FDR < 0.05 or Q < 0.05 were considered to be differentially expressed in edgeR and Cuffdiff, respectively. Prior to multiple testing correction 1353 differentially expressed genes were identified with a *p* < 0.05 in both programs, with 511 (37.7%) significant in both. After multiple testing correction, a total of 131 genes met criteria for differential expression in one of the analysis programs, with 6 identified in both. Here we have taken identification in either analysis program to represent differentially expressed genes. The multidimensional scaling plot was produced by the Limma plotMDS function, using the BCV method. The scatterplot shows the mean of normalised A and B replicates for both conditions.

## Additional files

Additional file 1: Figure S1. Principal component analysis. Principal component analysis of 4 RNA-seq replicates (A-D) performed with edgeR data in the Limma plotMDS function, using the BCV method. Each replicate is denoted with different colours, with the r1-r2 data as open circles. Expression data from replicates A-B group close together, while replicates C-D failed to cluster with the other data. (TIF 923 kb)

Additional file 2: Table S1. Total expression data from r1-r2 and r4 including replicates A-B. **Table S2.** Over-represented transcription factor binding sites in genes up-regulated in r1-2 and r4. (XLSX 2249 kb)
