# Supplementary figures and images for: Transcriptome profiling reveals expression signatures of cranial neural crest cells arising from different axial levels
Source: BMC Dev Biol. 2017 Apr 13;17:5. doi: 10.1186/s12861-017-0147-z (PMC5390458; doi:10.1186/s12861-017-0147-z)

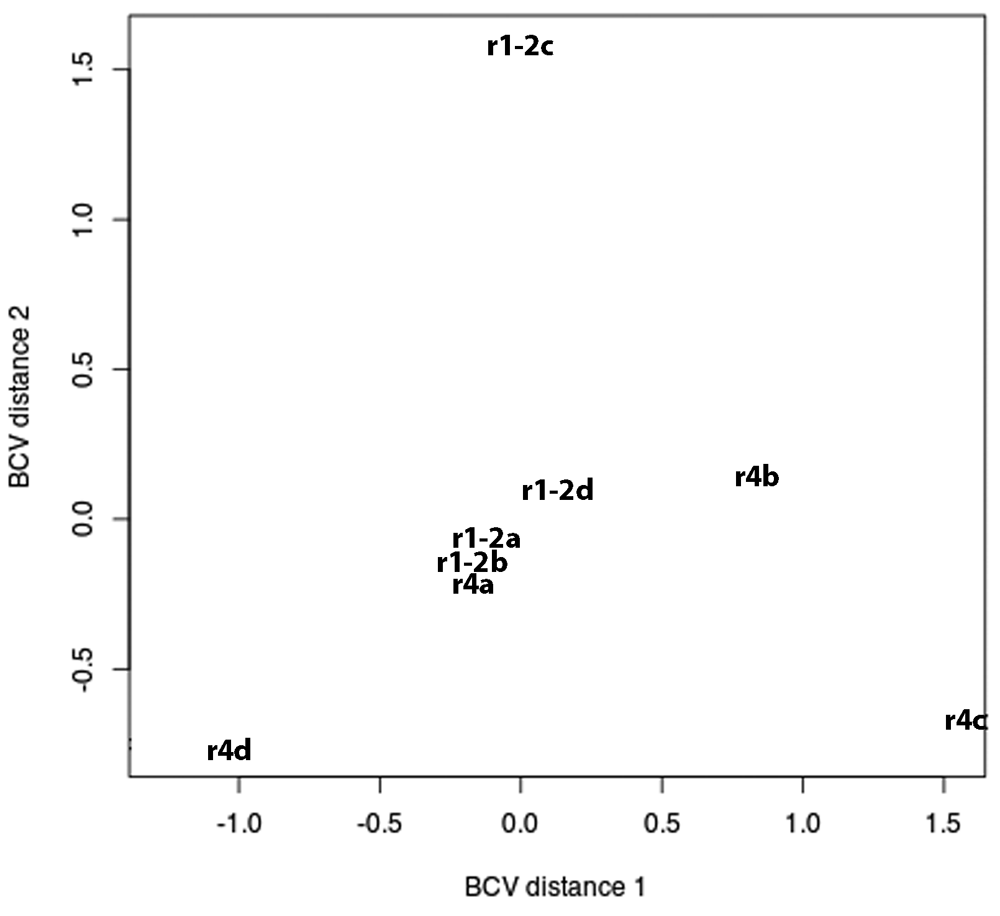

Supplement: Supplementary file 1 — Principal component analysis. Principal component analysis of 4 RNA-seq replicates (A-D) performed with edgeR data in the Limma plotMDS function, using the BCV method. Each replicate is denoted with different colours, with the r1-r2 data as open circles. Expression data from replicates A-B group close together, while replicates C-D failed to cluster with the other data. (TIF 923 kb) [file 12861_2017_147_MOESM1_ESM.tif]
